# Development of the Preterm Gut Microbiome in Twins at Risk of Necrotising Enterocolitis and Sepsis

**DOI:** 10.1371/journal.pone.0073465

**Published:** 2013-08-30

**Authors:** Christopher J. Stewart, Emma C. L. Marrs, Andrew Nelson, Clare Lanyon, John D. Perry, Nicholas D. Embleton, Stephen P. Cummings, Janet E. Berrington

**Affiliations:** 1 Faculty of Health and Life Sciences, University of Northumbria, Newcastle upon Tyne, United Kingdom; 2 Department of Microbiology, Freeman Hospital, Newcastle upon Tyne, United Kingdom; 3 Newcastle Neonatal Service, Royal Victoria Infirmary, Newcastle upon Tyne, United Kingdom; Instutite of Agrochemistry and Food Technology, Spain

## Abstract

The preterm gut microbiome is a complex dynamic community influenced by genetic and environmental factors and is implicated in the pathogenesis of necrotising enterocolitis (NEC) and sepsis. We aimed to explore the longitudinal development of the gut microbiome in preterm twins to determine how shared environmental and genetic factors may influence temporal changes and compared this to the expressed breast milk (EBM) microbiome. Stool samples (*n* = 173) from 27 infants (12 twin pairs and 1 triplet set) and EBM (*n* = 18) from 4 mothers were collected longitudinally. All samples underwent PCR-DGGE (denaturing gradient gel electrophoresis) analysis and a selected subset underwent 454 pyrosequencing. Stool and EBM shared a core microbiome dominated by Enterobacteriaceae, Enterococcaceae, and Staphylococcaceae. The gut microbiome showed greater similarity between siblings compared to unrelated individuals. Pyrosequencing revealed a reduction in diversity and increasing dominance of *Escherichia* sp. preceding NEC that was not observed in the healthy twin. Antibiotic treatment had a substantial effect on the gut microbiome, reducing *Escherichia* sp. and increasing other Enterobacteriaceae.

This study demonstrates related preterm twins share similar gut microbiome development, even within the complex environment of neonatal intensive care. This is likely a result of shared genetic and immunomodulatory factors as well as exposure to the same maternal microbiome during birth, skin contact and exposure to EBM. Environmental factors including antibiotic exposure and feeding are additional significant determinants of community structure, regardless of host genetics.

## Introduction

The gut microbiome is crucial to both health (immunomodulation/protection, nutrition and metabolism) and disease (inflammation, diabetes, obesity and allergy) [1]. Due to the complexities of both the microbial community and factors that affect it, exploring individual variables (including diet, medical interventions/exposures and genetic components) is challenging. Studying twins or higher order multiples may provide unique insights. Healthy twins have been shown to develop a comparable gut microbiome after term birth [2], in childhood [3] and into adulthood [4], suggesting genetic or shared environmental factors shape the gut community. However, there is currently a lack of research exploring the temporal changes of the gut microbiome in preterm twins.

Preterm neonates provide a unique cohort to study the gut microbiome due to intensive care practises and the susceptibility of these infants to disease, such as necrotising enterocolitis (NEC) and sepsis, resulting in differential microbiome development compared to term infants [5]. NEC is an inflammatory disease process of the intestine with a pathogenesis strongly linked to immune dysregulation, enteral feeding, and abnormal bacterial colonisation [5]. Changes in the microbial community have been associated with NEC and sepsis in unrelated preterm neonates [6,7]. Studying twins with NEC may help elucidate the role of specific exposures that may be key to reducing incidence of these diseases. Despite previous studies implicating dysbiosis of the gut microbiome in disease development, no single causative agent has been convincingly identified as a biomarker of NEC. A previous study of 20 infants, including 4 sets of discordant twin pairs (one with NEC, one without) confirmed that the communities in the bowels of NEC infants were different to healthy controls [8]. However, sampling was inconsistent and was not longitudinal, thus any temporal changes in microbial community dynamics that may have been associated with the pathophysiology of NEC were not captured.

For term infants vaginal delivery and receipt of maternal breast milk are key factors that facilitate the development of a ‘healthy’ microbiome. Breast milk contains many immunomodulatory factors that support growth and prevent infection including lysozyme, lactoferrin, and oligosaccharides as well as live bacteria which regulate host-microbe interactions [9] and modify infant gut microbiome development [10]. Preterm infants are less likely to experience vaginal birth or only receive breast milk feeds and are more likely to experience many medical interventions that affect the microbiome. To what extent the infant gut microbiome reflects the maternal breast milk microbiome is currently unclear.

In a twin cohort we aimed to explore the longitudinal development of the gut bacterial community after preterm birth by analysis of stool and expressed breast milk (EBM). In addition we focused on the development of NEC in one set of twins with regular longitudinal sampling and where only one infant was diagnosed with NEC.

## Materials and Methods

### Patients and samples

Preterm infants (<32 weeks gestation) from multiple births cared for in the Royal Victoria Infirmary (Newcastle upon Tyne, UK) had stool collected between April 2010 and February 2012. Stool samples (*n* = 173) were salvaged directly from the nappy from 12 sets of twins and 1 set of triplets (*n* = 27) from birth until discharge. EBM (*n* = 18) from 4 mothers was also analysed as EBM samples were only salvaged toward to latter stages of the study and often insufficient volume was acquired. EBM samples were obtained from the remaining liquid in the syringe after continual feed to the neonate through a catheter tube. All samples were frozen at -20°C within 24 hours. This unit has standardised feeding practices, antibiotic and antifungal use. Fluconazole is used as antifungal prophylaxis if <26 weeks/<1kg and in babies developing NEC. Antibiotics, specifically Penicillin and Gentamycin, were administered to all infants following birth and stopped at 48 hours unless proven infection. Clinical information was obtained from patient notes: NEC was confirmed independently by two experienced neonatal clinicians (JEB/NDE): sepsis was defined by positive blood culture, along with antibiotic treatment for a minimum of 5 days and clinical signs suggestive of infection.

### DNA Extraction

Nucleic acid extraction of stool was carried out on 100 mg of sample using the PowerLyzer™ PowerSoil® DNA Isolation Kit (MoBio, CA, USA). Nucleic acid extraction of EBM was performed on 1.8 mL of EBM using the Powerfood™ Microbial DNA Isolation Kit (MoBio, CA, USA). Both kits were used in accordance with the manufacturer’s instructions.

### PCR-DGGE

PCR amplification targeted the V3 region within the bacterial 16S rRNA gene and was performed with universal primers for total bacterial amplification; V3F-GC (5'-CGCCCGCCGCGCGCGGCGGGCGGGGCGGGGGCACGGGGGGCCTACGGGAGGCAGCAG-3') and V3R (5'-ATTACCGCGGCTGCTGG-3') as described by Muyzer et al. [11], according to the methods of Baxter and Cummings [12].

DGGE (denaturing gradient gel electrophoresis) analysis was performed using the D-Code DGGE system (Bio-Rad, CA, USA) and selected DGGE bands (n=17) underwent excision and sequencing as previously described [13] (Table S1). A ladder of amplicons derived from pure culture organisms was loaded on each DGGE gel for multiple gel alignment. The analysis of the DGGE banding patterns employed TotalLab Phoretix 1D and 1D Pro software (Nonlinear Dynamics, Newcastle upon Tyne, UK). Sequencing was carried out on an ABI 3730XL sequencer (Eurofins MWG Operon, London, UK) and BLASTn was used to determine the closest sequence homologies. DGGE sequence data was deposited to the National Center for Biotechnology Information GenBank under the accession numbers KC686617-KC686622. Analysis of sequence data was carried out at the genus level where possible. As the sequences generated were based on a ~200 bp fragment of the 16S rRNA gene, robust species level classification is not feasible.

### 454 Pyrosequencing

A subset of key samples (Stool = 36EBM = 5) was selected for metagenomic sequencing: primarily EBM and stool from the triplets (patients 145/147/148) and a discordant twin pair where one developed NEC (patients 139/140). Although other twin sets in the study were discordant for NEC and/or sepsis, patients 139/140 provided the most robust sampling pre, during, and post disease diagnosis. 454 pyrosequencing was carried out using the bifidobacteria-optimised primer set 357F (5'-CTCCTACGGGAGGCAGCAGAN-3') and 926Rb (5'-CCGTCAATTYMTTTRAGT-3') [14]. The DNA sequencing was performed using the 454 Genome Sequencer FLX Titanium platform (Roche, IN, USA) by the Research and Testing Laboratory (RTL, TX, USA) using previously described methods [15].

The raw sequencing reads were quality filtered in QIIME 1.6.0 [16] using the split-library.py script with the following criteria: 1) exact matches to bar code tags; 2) no ambiguous bases; 3) maximum of 5 mismatches to primer sequence; 4) read-lengths between 200–700 base pairs (bp); 5) average quality score of >25 in a sliding window of 50 bp. Remaining high quality sequences were clustered into operational taxonomic units (OTUs) at 97% similarity using UCLUST [17]. Representative sequences for each OTU were aligned using PyNAST [18] and taxonomic identities were assigned using RDP-classifier (version 2.2) [19] with 50% as confidence value threshold. Detection of potentially chimeric sequences was performed using ChimeraSlayer [20] and chimeric sequences were removed from downstream analysis prior to tree building using FastTree [21]. Sequences were deposited in MG-RAST under the accession numbers 4516545.3-4516585.3

### Statistical analysis

The DGGE profiles obtained from both stool and EBM samples were analysed using multivariate partial least squares discriminant analysis (PLS-DA) using SIMCA 13.0 (Umetrics, Stockholm, Sweden) [22]. To check that data was adhering to multivariate normalities, Hotelling’s *T* ^2^ tolerance limits were calculated and set at 0.95. Shannon diversity indices (H'), a diversity index giving a measure of both species richness and the evenness of their abundance, was calculated using the PAST package [23].

The sequence reads generated using 454 pyrosequencing were analysed using weighted UniFrac [24] and visualised using principal coordinate analysis (PCoA). Rarefaction curves were produced to 5000 sequences for all samples (Figure S1). Martin’s P-test of significance [25] was applied to determine if significant differences were occurring in the profiles between and within patients. For all statistical analysis, a probability (*P*) value of <0.05 was deemed to be statistically significant.

### Ethics Statement

Initial collection was part of routine service and all samples were collected during the course of normal treatment. Ethical approval was obtained from the County Durham and Tees Valley Research Ethics Committee to include molecular techniques in August 2010. For all infant stool samples, prospective parental informed consent was documented at the point of donation to have the samples stored for research purpose. For EBM, informed consent was documented at the point of donation to have the samples stored for research purposes from March 2011.

## Results

### Patients

27 patients (12 twin pairs 1 triplet set) contributed to the study: five developed definite NEC and five developed sepsis (two of whom also had NEC). Demographic information is summarised in Table 1 and a detailed version based on single samples can be found in the supplementary information (Table S2). No infant received supplemental probiotics.

**Table 1:** Summary of patient demographics.

**Patient**	**Delivery Mode**	**Gestation (weeks)**	**Birth Weight (g)**	**Sex**	**Chorionicity**	**EBM ever**	**NEC**	**NEC Diagnsis (DOL^a^)**	**BC^b^+**	**BC^b^ (DOL^a^)**	**Organism**	**Number of samples collected**
22	Caesarean	27	870	M	DD^c^	Y	N		N			2
23	Caesarean	27	790	F	DD^c^	Y	N		N			6
28	Caesarean	28	1250	M	MM^d^	Y	N		N			6
29	Caesarean	28	1180	M	MM^d^	Y	Y	17	N			1
39	Caesarean	25	780	M	DD^c^	N	N		Y	15	CoNS^g^	8
41	Caesarean	25	820	M	DD^c^	N	N		Y	15	*S. aureus*	8
46	Caesarean	26	830	M	DD^c^	Y	N		N			6
47	Vaginal	26	760	F	DD^c^	Y	Y	45	Y	03/08	CoNS^G^ / *Pseudomonas* sp.	5
51	Caesarean	27	1060	M	DD^c^	Y	Y	16	Y	17/56	CoNS^g^ / CoNS^g^	3
55	Caesarean	27	1100	M	DD^c^	Y	N		N			2
68	Vaginal	26	760	M	DD^c^	Y	N		N			4
70	Vaginal	26	860	M	DD^c^	Y	N		Y	40	CoNS^g^	2
92	Caesarean	25	740	M	DD^c^	Y	N		Y	8	*K. pneumoniae*	1
93	Caesarean	25	670	M	DD^c^	Y	N		N			5
100	Caesarean	27	1050	M	MD^d^	Y	N		N			6
101	Caesarean	27	910	M	MD^d^	Y	N		N			5
112	Vaginal	25	700	F	DD^c^	Y	N		N			12
113	Vaginal	25	680	M	DD^c^	Y	N		Y	57	CoNS^g^	7
135	Caesarean	29	910	F	MM^e^ (TTTS)^c^	N	N		Y	54	*S. aureus + K. pneumoniae*	8
136	Caesarean	29	1275	F	MM^e^ (TTTS)^c^	N	N		N			3
139	Caesarean	30	1470	M	DD^c^	Y	Y	28	N			25
140	Caesarean	30	1455	F	DD^c^	Y	N		N			20
145	Caesarean	31	990	M	MM^e^	Y	N		N			10
148	Caesarean	31	1455	M	MM^e^	Y	N		N			4
147	Caesarean	31	1540	M	DD^c^	Y	N		N			5
151	Vaginal	27	1020	F	DD^c^	Y	N		N			4
154	Vaginal	27	1060	M	DD^c^	Y	Y	21	N			5

^a^ Day of life.

^b^ Blood culture.

^c^ Dichorionic diamniotic.

^d^ Monochorionic diamniotic.

^e^ Monochorionic monoamniotic.

^f^ TTTS – Twin to Twin Transfusion Syndrome.

^g^ Coagulase negative *Staphylococcus* .

### Stool profiles

**Figure 1:**
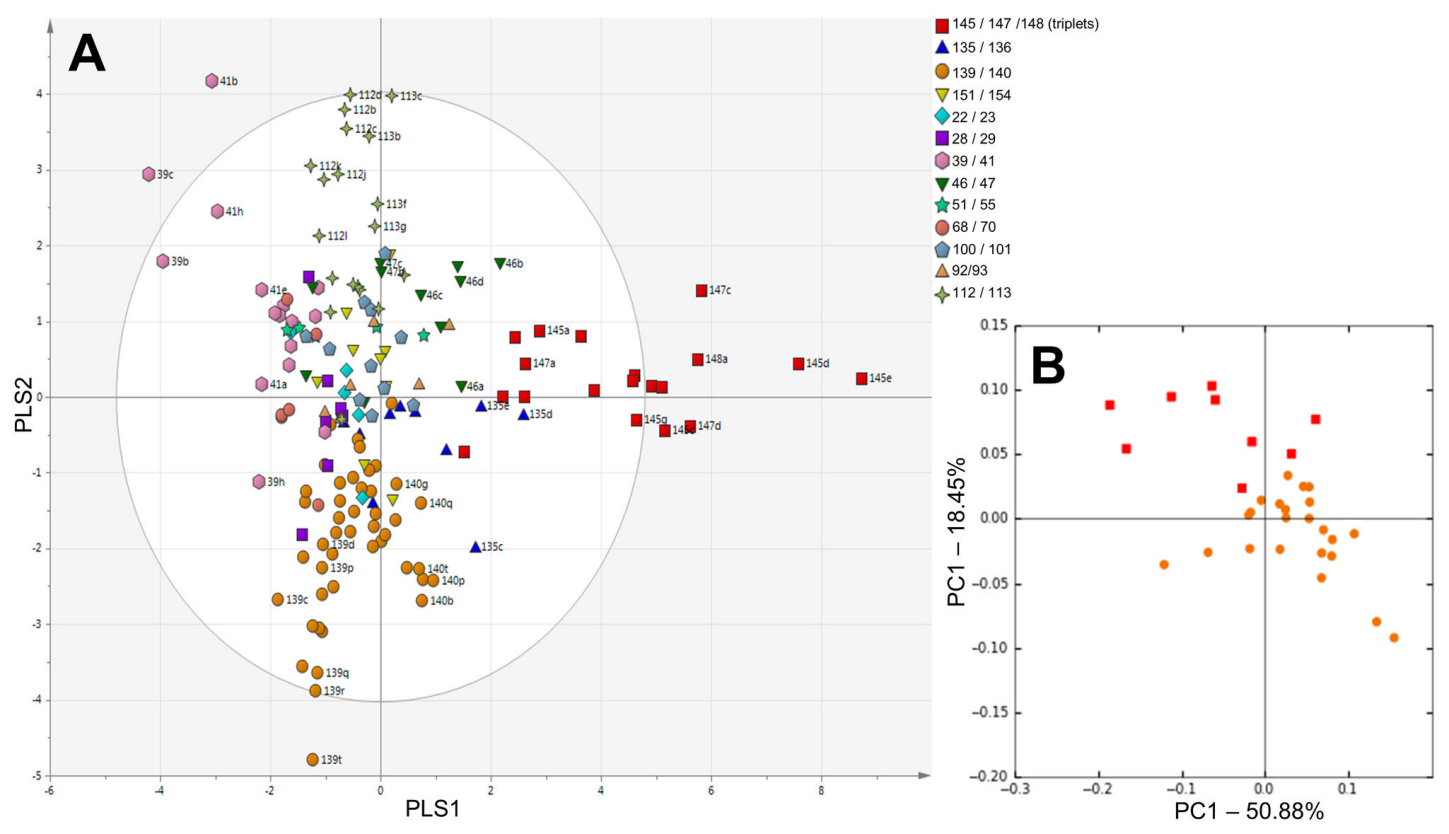
Community profiles of gut (stool) microbiome from preterm multiples. Subjects are symbolised based on related multiples. a) Partial least squares discriminant analysis (PLS-DA) based on DGGE data of all stool samples. The ellipse indicates Hotellings *T* ^2^ range, at 95% confidence. Selected sample labels removed for clarity. b) Weighted UniFrac PCoA based on pyrosequencing data at genus level, generated from a subset of stool samples.

PLS-DA of all samples from all patients based on DGGE data demonstrated twin pairs had comparable profiles which were distinct from unrelated individuals (Figure 1A). Samples which fell outside the ellipse (indicating Hotellings *T* ^2^ range, at 95% confidence) belonged to the triplets or were associated with late onset infection. Specifically, this comprised sample 139t collected one day post NEC diagnosis as well as samples 39c and 41b collected three days prior and on the day of sepsis diagnosis, respectively. Based on DGGE analysis of the cohort, a subset of longitudinal samples from the triplets (represented by red squares) and twin pair discordant for NEC (represented by orange circles) which showed significant variation in community development were selected for pyrosequencing. The pyrosequencing data was analysed at the genus level using weighted UniFrac. In accordance with the DGGE data, samples grouped with their related twin, showing high intra-sample similarities in the development of the gut microbiome (Figure 1B). This is reflected in the bar plots which show each set of twins and the triplets developed a distinct gut microbiome (Figure S2). Proteobacteria and Firmicutes dominated samples in both DGGE and pyrosequencing. From pyrosequencing, 5 OTUs were found in the core microbiome (present in over 85% of samples) in stools from the families Enterobacteriaceae, Enterococcaceae, and Staphylococcaceae.

### Comparison of breast milk with respective stool samples

**Figure 2:**
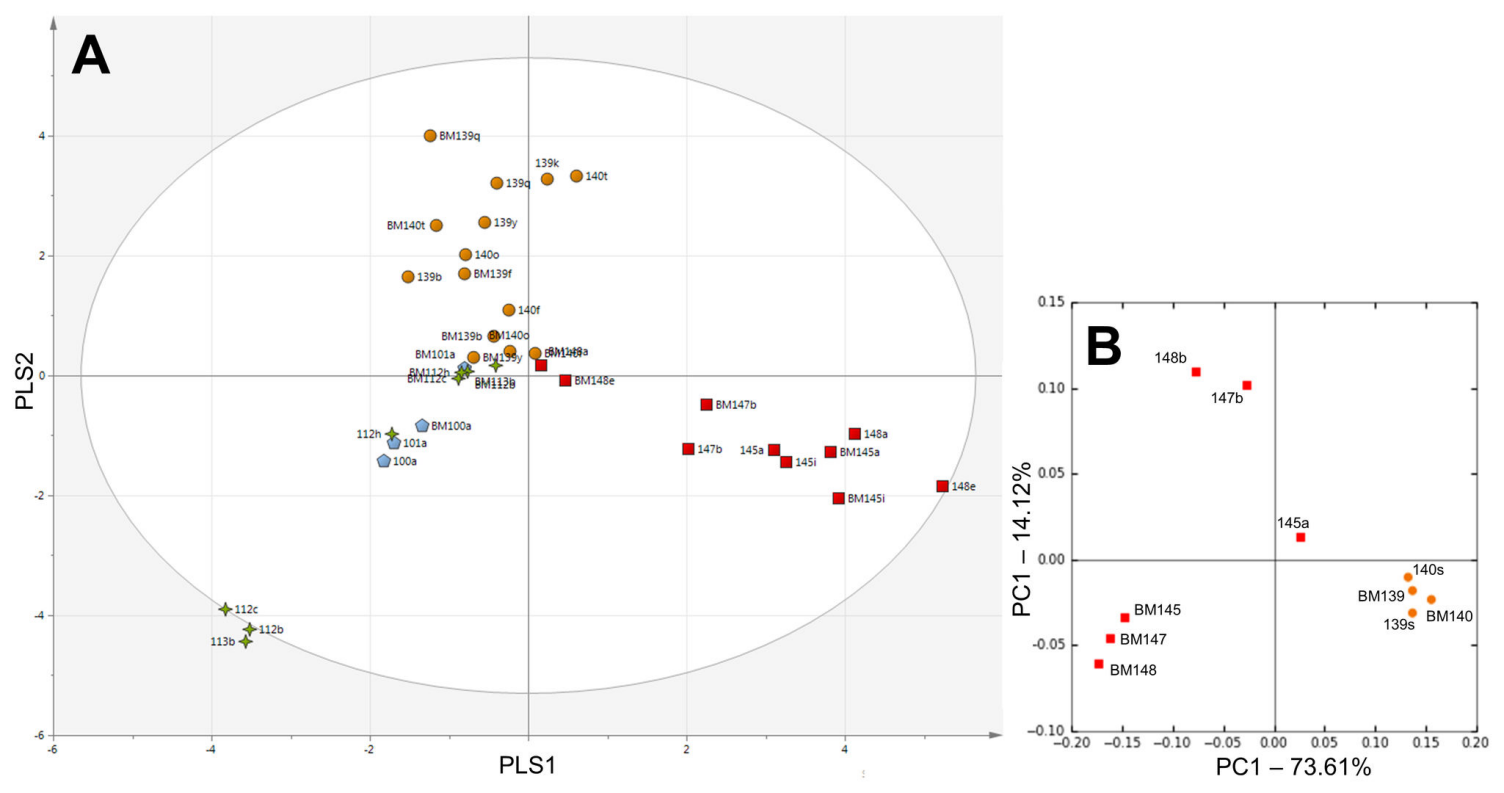
Comparison of breast milk with respective stool profiles. Subjects are symbolised based on related multiples. a) Partial least squares discriminant analysis (PLS-DA) based on DGGE data of all breast milk (EBM) samples matched to respective stools. The ellipse indicates Hotellings *T* ^2^ range, at 95% confidence. b) Weighted UniFrac PCoA based on pyrosequencing data at genus level, generated from a subset of EBM and respective stool samples.

The DGGE profiles of all EBM samples were compared with the respective stool sample. EBM samples showed a relative lack of diversity making them cluster near the origin: despite this PLS-DA did reveal EBM samples clustered with the stool samples of the respective set of multiples (Figure 2A). There are cases where different EBM samples from the same mother cluster separately showing the EBM microbiome was not stable (e.g. BM148e / BM147b / BM145a / BM145i or BM139b / BM139f / BM139q).

EBM from the triplet set and twin set (patients 139/140) also underwent pyrosequencing. EBM samples from each mother showed high intra-sample similarity (Figure 2B). For twins 139/140 the EBM profiles (BM139/BM140) clustered closely with the respective stool (139s/140s). This clustering was less robust in the triplet set although triplet EBM (BM145/BM147/BM148) was still more similar to triplet stool (145a/147b/148b) than stool of other infants. The connection of EBM samples with stool can be visualised in the bar plot (Figure S2). Three OTUs were found in the core microbiome in EBM and, like in the stool core microbiome, were from families Enterobacteriaceae, Enterococcaceae, and Staphylococcaceae.

### Bacterial community development in twin set 139/140

**Figure 3:**
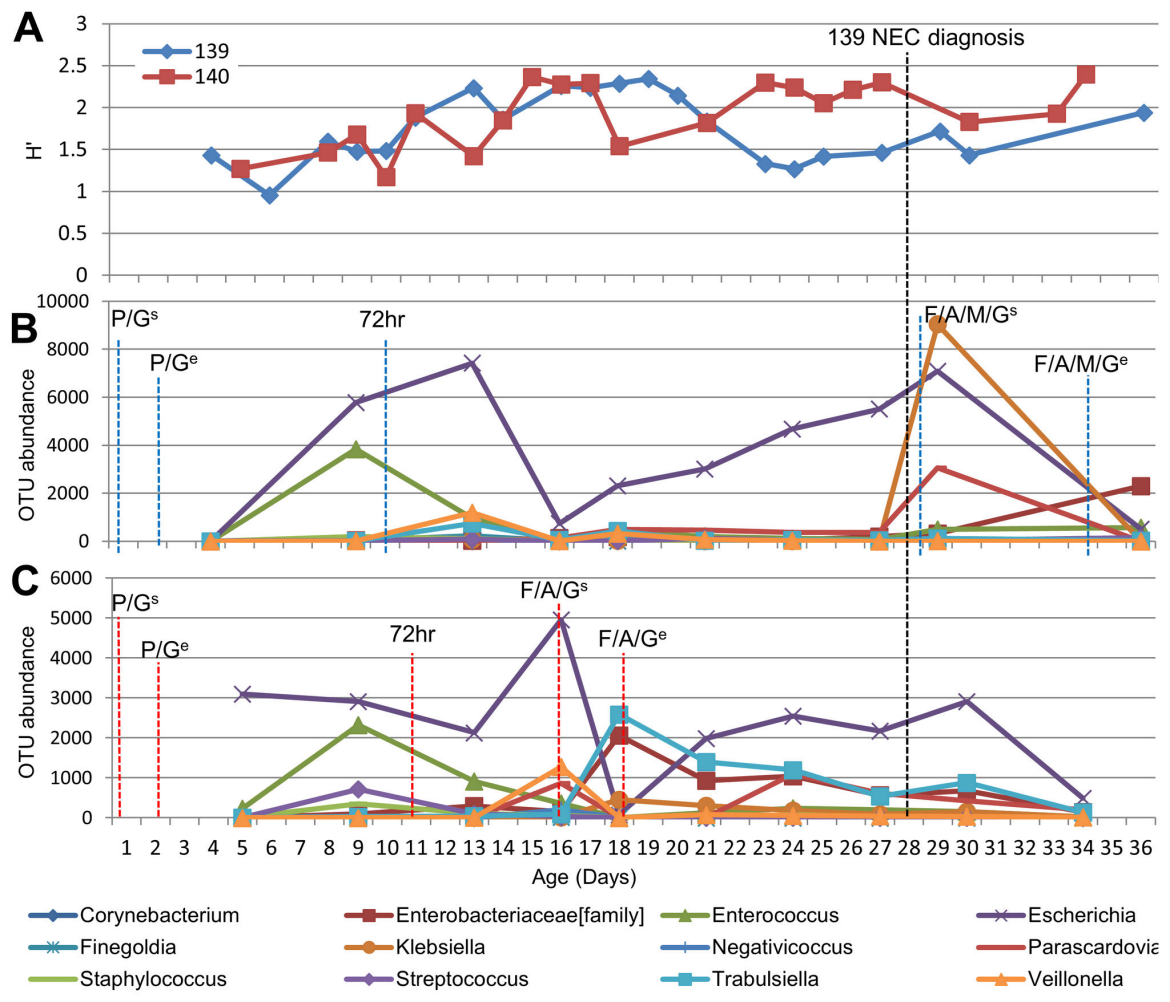
Development of gut microbiome in twin pair 139/140 mapped to life events. P – Penicillin, G – Gentamicin, F- Fluctoxacillin, A - Amoxicillin, M – Metronidazole, ^s^ –Start of antibiotics, ^e^ – End of antibiotics, 72hr – full enteral feed (at least 150 ml/kg/day) sustained for 72 hours. a) Shannon Diversity indices (H') of twin pair based on DGGE data b) Turnover of the most prevalent bacterial OTUs throughout the first 36 days of life in twin 139 where antibiotics were prescribed for NEC. c) Turnover of most prevalent bacterial OTUs throughout the first 34 days of life in twin 140 where antibiotics were prescribed due to pyrexia (fever).

A reduction in diversity was observed in patient 139 at least 5 days before NEC was apparent clinically (Figure 3A), which was not shared by the twin. A less pronounced reduction in diversity did occur in the control twin (140) earlier in development (day 18) that coincided with antibiotic administration and diversity was quickly re-established when antibiotics ceased. A similar trend was observed when the H' of the pyrosequencing data was performed (data not shown).

Pyrosequencing of matched twin samples (full profiles in the bar plot; Figure S2) allowed the 12 most abundant OTUs to be incorporated into the analysis (Figure 3). These results are in accordance with the DGGE data and allow greater detail about specific contributors to the overall diversity to be observed. While community structure in the twins was initially comparable, twin 139 showed reducing diversity with an increasing abundance of *Escherichia* sp., before NEC diagnosis on day 28 (Figure 3B). Conversely, in the sibling there is an increase in diversity and *Escherichia* sp. was present in much lower abundance (Figure 3C). After antibiotic receipt (day 16 in twin 140 and day 28 in twin 139) both twins demonstrate reduced *Escherichia* sp. abundance and an increased abundance of other Enterobacteriaceae, rapidly reversing in twin 140 on antibiotic cessation. In twin 139 NEC and subsequent antibiotic treatment significantly (*P*=0.028) altered the bacterial community in comparison to its sibling, with a new notable bloom in *Klebsiella* sp. and a smaller increase in *Parascardovia* sp. (family – Bifidobacteriaceae).

## Discussion

In this study, we hypothesised that the bacterial community in related twins and a set of triplets would be comparable and reflect maternal EBM community. In cases of twin pairs discordant for NEC we hypothesized that they would show differences in microbial community development prior to NEC onset.

We have demonstrated in a preterm population with multiple clinical exposures that the development of the gut microbiome is more similar between genetically related individuals than between other preterm infants. However, due to similar environmental exposures encompassed by related individuals, this may not be a direct result of host genetics. Interestingly, community structure was similar for all triplets even though two (145/148) were monochorionic monoamniotic and the other (147) was dichorionic. This suggests that shared factors (genetic or environmental) are important in determining the gut microbiome even in an environment with many complex variable factors that also affect community development [2,26].

Proteobacteria and Firmicutes dominated the gut microbiome over the initial weeks of life as previously reported [27]. Stool and EBM shared a core microbiome of the families Enterobacteriaceae, Enterococcaceae, and Staphylococcaceae. Others describing the EBM microbiome noted the presence of Streptococcaceae which in was present in low abundance in our maternal cohort [28,29]. We also detected a low abundance of bifidobacteria and lactobacilli in stool and EBM despite their reported prevalence by others [10,28,30]. This may be attributable to differences among subjects, organisms residing in the unit, and detection by differing techniques [29], but occurred despite the use of optimised universal primers in pyrosequencing designed to facilitate the detection of Bifidobacteria [14]. Notably, the cohort in this study is significantly preterm which can result in delayed colonisation with bifidobacteria; geographical or demographic differences may also be attributable to the low prevalence of this organism [2,7]. The milk microbiome was not stable throughout lactation [31] and EBM appeared to be an on-going source of new bacteria contributing to the dynamic nature of the gut microbiome. Furthermore, genera which typically reside on adult skin including *Staphylococcus* , *Corynebacterium* , and *Propionibacterium* were found in high abundance in the gut microbiome suggesting skin contact may be an important source of bacterial acquisition, even within the nursery environment [32].

The importance of the gut microbiome in disease is increasingly recognised, despite a lack of consistent causative agent between studies. In a focused temporal exploration from a twin pair discordant for NEC, we showed clear changes attributable to antibiotic exposure and NEC development, with effects on the dominance of *Escherichia* sp. and the abundance of other Enterobacteriaceae sp. [33]. The significantly different community observed in sample 139t is likely attributable to a temporary bloom in *Klebsiella* sp. following NEC diagnosis, which was reduced in the subsequent sample following broad spectrum antibiotic administration.

A twin study methodology has been utilised in other inflammatory bowel diseases such as ulcerative colitis and Crohn’s disease, but there are few comparable studies in NEC. Interestingly, analogous to NEC, dysbiosis is a major factor in the pathogenesis of these diseases, consistent with a lack of a single causative agent [34]. Specifically, a decreased diversity in the gut microbiome of ulcerative colitis and Crohn’s disease patients compared to healthy controls has been noted [8,35], and multiple studies report increased abundances of Proteobacteria, particularly *Escherichia* sp. [36,37]. This increase in Proteobacteria is an emerging theme in NEC development [6,13], but is probably one of several factors needed for NEC development. *Escherichia* spp. are reported pathogens [38] and the association of this genus with inflammatory mediated disease warrants further investigation.

Our use of molecular approaches for community profiling circumvents the known limitations of culturing human gut species [39]. We utilised cost effective DGGE to educate sample selection for 454 pyrosequencing. Studies on preterm infants, especially of multiple births, are difficult due to the exclusivity of the cohort and thus only 27 patients could be included. With the exception of the twin set 139/140, twins discordant for disease often lacked informative longitudinal samples; in large due to the withdrawal of feeds resulting in reduced excrement. Although the number and timing of samples collected from each set of twins was generally comparable, varying numbers of samples were collected between twin sets which may bias some analysis. Overall, the data generated using DGGE and pyrosequencing were in agreement, perhaps due to primer sets encompassing the V3 hypervariable region of the 16S rRNA genome in both analyses. Pyrosequencing allows larger fragments to be amplified allowing the use of bifidobacteria-optimised universal primers. Despite this, bifidobacteria was not found to be a prevalent genus [2,7] which requires further study due to the potential use of this taxa as probiotic in therapeutic intervention [40].

In summary, this study represents a unique temporal analysis of the gut microbiome in preterm twins, cared for within the complex environment of neonatal intensive care. Although twins discordant for late onset infection showed differences in gut microbiome development, overall, related infants harboured bacterial communities more similar to each other than nonrelated infants. As well as shared genetic and immunomodulatory factors, this is likely a result of exposure to the same maternal microbiota during birth, skin contact and exposure to EBM. We have also shown that other environmental factors, particularly antibiotic exposure, have additional significant effects on the gut microbiome in genetically related infants. Antibiotics have been shown to alter the gut microbiome in term [2] and adult populations [41] and the exact role of individual antibiotics in altering the preterm gut microbiome warrants further investigation. We have further noted potential concurrence between community changes associated with other inflammatory mediated diseases, such as ulcerative colitis and Crohn’s disease, and those increasingly reported to occur in NEC.

## Supporting Information

Figure S1 **Rarefactions curves produced in QIIME to 5000 sequences comparing all samples (stool and expressed breast milk).**
(TIF)

Figure S2 **Order level bar plot of all samples (stool and expressed breast milk) which underwent 454 pyrosequencing.**
(TIF)

Table S1 **DGGE band identities and prevalence.**
(XLSX)

Table S2 **Complete table of patient demographics.**
(XLSX)
