# Supplementary figures and images for: Development of the Preterm Gut Microbiome in Twins at Risk of Necrotising Enterocolitis and Sepsis
Source: PLoS One. 2013 Aug 30;8(8):e73465. doi: 10.1371/journal.pone.0073465 (PMC3758342; doi:10.1371/journal.pone.0073465)

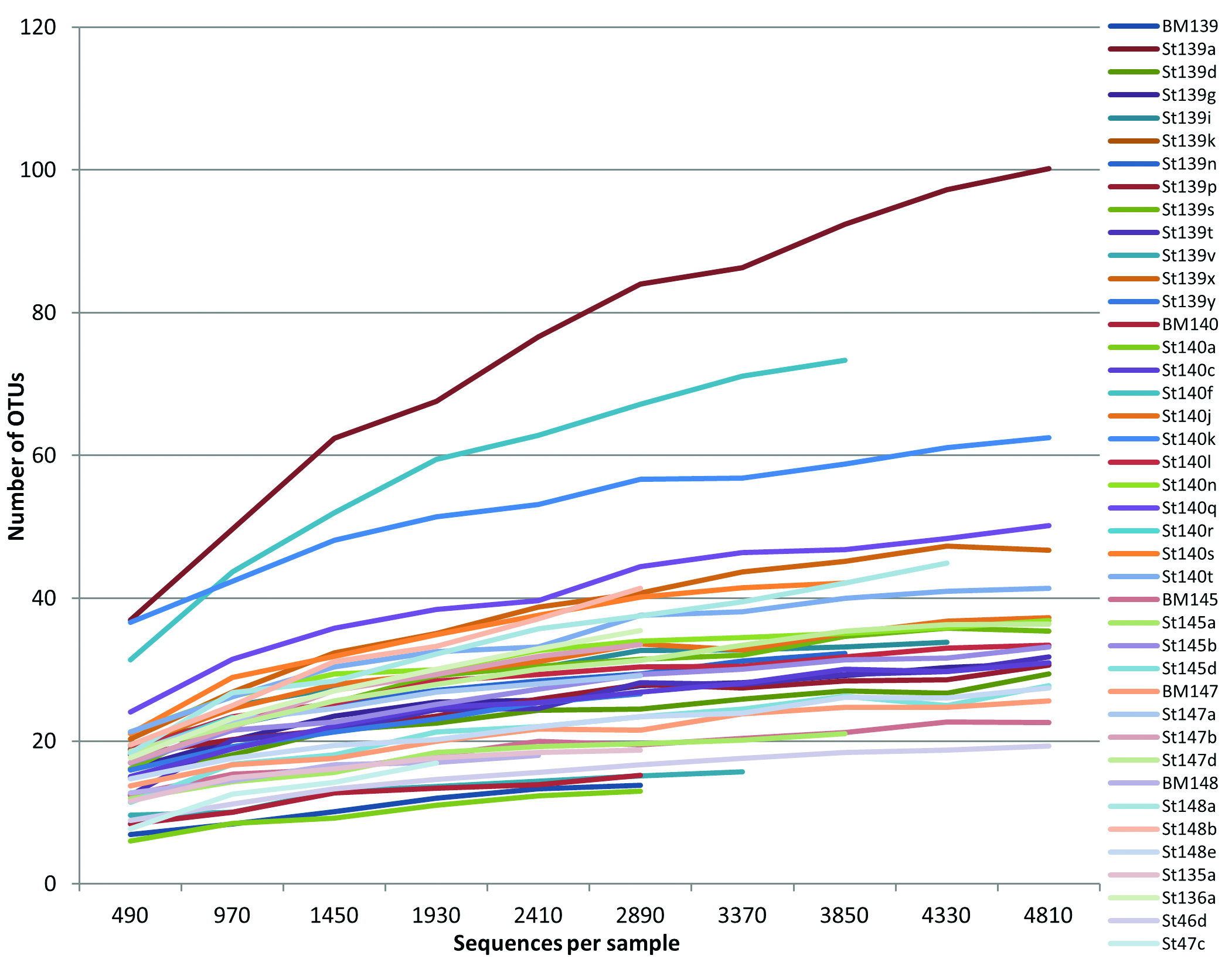

Supplement: Figure S1 — Rarefactions curves produced in QIIME to 5000 sequences comparing all samples (stool and expressed breast milk). (TIF) [file pone.0073465.s001.tif]

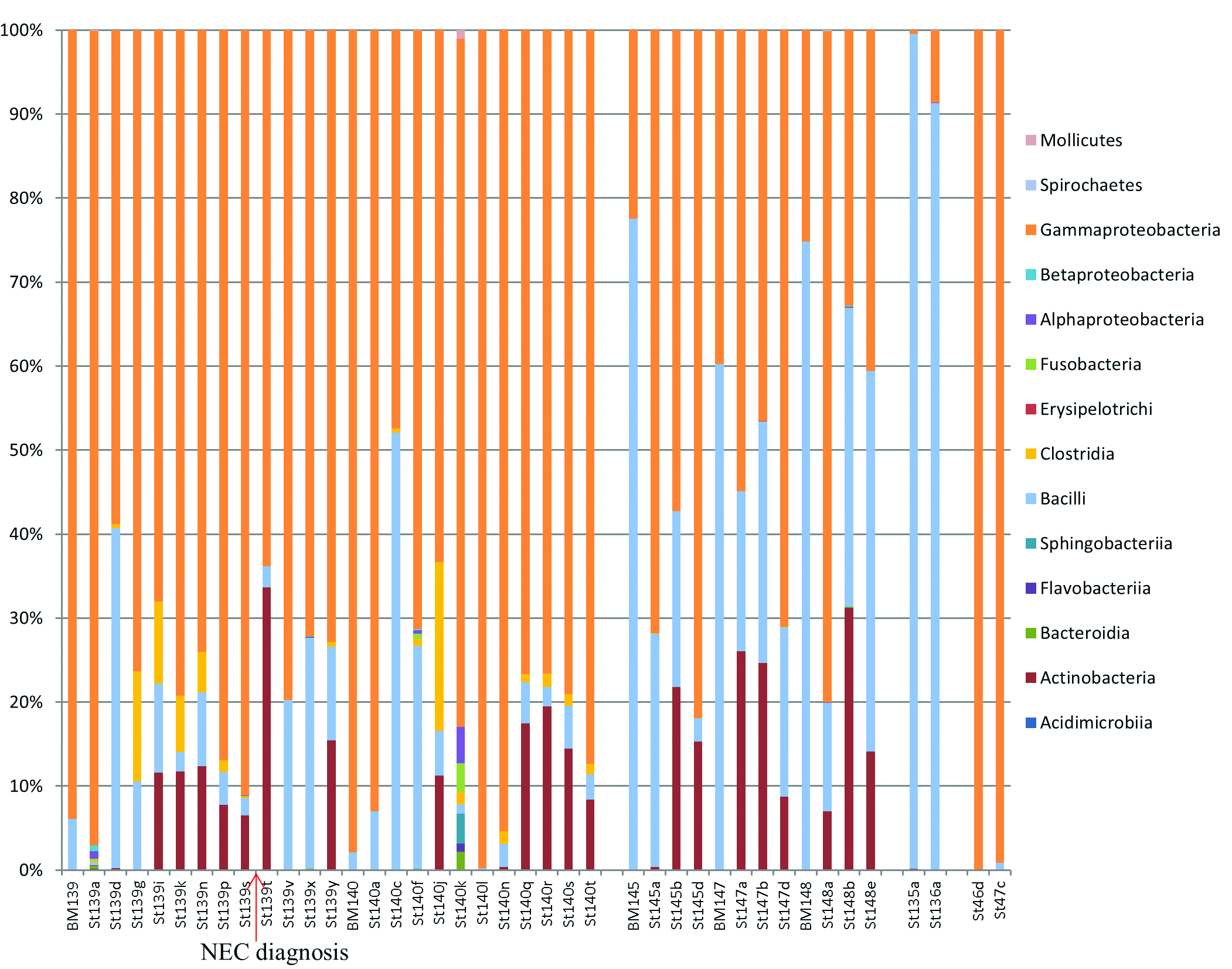

Supplement: Figure S2 — Order level bar plot of all samples (stool and expressed breast milk) which underwent 454 pyrosequencing. (TIF) [file pone.0073465.s002.tif]
